# A randomised phase II study of modified FOLFIRI.3 *vs* modified FOLFOX as second-line therapy in patients with gemcitabine-refractory advanced pancreatic cancer

**DOI:** 10.1038/sj.bjc.6605374

**Published:** 2009-10-13

**Authors:** C Yoo, J Y Hwang, J-E Kim, T W Kim, J S Lee, D H Park, S S Lee, D W Seo, S K Lee, M-H Kim, D J Han, S C Kim, J-L Lee

**Affiliations:** 1 Department of Internal Medicine, Asan Medical Center, University of Ulsan College of Medicine, Seoul, Korea; 2 Department of Oncology, Asan Medical Center, University of Ulsan College of Medicine, Seoul, Korea; 3 Department of Gastroenterology, Asan Medical Center, University of Ulsan College of Medicine, Seoul, Korea; 4 Department of Surgery, Asan Medical Center, University of Ulsan College of Medicine, Seoul, Korea

**Keywords:** second-line chemotherapy, pancreatic cancer, irinotecan, oxaliplatin, gemcitabine

## Abstract

**Background:**

Only a few clinical trials have been conducted in patients with advanced pancreatic cancer after failure of first-line gemcitabine-based chemotherapy. Therefore, there is no current consensus on the treatment of these patients. We conducted a randomised phase II study of the modified FOLFIRI.3 (mFOLFIRI.3; a regimen combining 5-fluorouracil (5-FU), folinic acid, and irinotecan) and modified FOLFOX (mFOLFOX; a regimen combining folinic acid, 5-FU, and oxaliplatin) regimens as second-line treatments in patients with gemcitabine-refractory pancreatic cancer.

**Methods:**

The primary end point was the 6-month overall survival rate. The mFOlFIRI.3 regimen consisted of irinotecan (70 mg m^−2^; days 1 and 3), leucovorin (400 mg m^−2^; day 1), and 5-FU (2000 mg m^−2^; days 1 and 2) every 2 weeks. The mFOLFOX regimen was composed of oxaliplatin (85 mg m^−2^; day 1), leucovorin (400 mg m^−2^; day 1), and 5-FU (2000 mg m^−2^; days 1 and 2) every 2 weeks.

**Results:**

Sixty-one patients were randomised to mFOLFIRI.3 (*n*=31) or mFOLFOX (*n*=30) regimen. The six-month survival rates were 27% (95% confidence interval (CI)=13–46%) and 30% (95% CI=15–49%), respectively. The median overall survival periods were 16.6 and 14.9 weeks, respectively. Disease control was achieved in 23% (95% CI=10–42%) and 17% patients (95% CI=6–35%), respectively. The number of patients with at least one grade 3/4 toxicity was identical (11 patients, 38%) in both groups: neutropenia (7 patients under mFOLFIRI.3 regimen *vs* 6 patients under mFOLFOX regimen), asthaenia (1 *vs* 4), vomiting (3 in both), diarrhoea (2 *vs* 0), and mucositis (1 *vs* 2).

**Conclusion:**

Both mFOLFIRI.3 and mFOLFOX regimens were tolerated with manageable toxicity, offering modest activities as second-line treatments for patients with advanced pancreatic cancer, previously treated with gemcitabine.

Pancreatic cancer accounts for 3% of all cancers, but is the fifth leading cause of cancer death in Western countries (Yeo *et al*, 2005). At the time of diagnosis, approximately half of the patients have metastases, and the median survival time barely exceeds 6 months, whereas approximately one-third of patients diagnosed with locally advanced disease have median survival times ranging between 6 and 9 months. Thus, a small proportion of patients are eligible for surgery, the only curative treatment option, at diagnosis (Bilimoria *et al*, 2007). Even with surgery, prognosis remains poor; the 5-year overall survival was only 23.4% for patients undergoing pancreatectomy (Sener *et al*, 1999).

Although 5-fluorouracil (5-FU)-based chemotherapy has been reported to be superior to best supportive care alone (Palmer *et al*, 1994; Glimelius *et al*, 1996), and a pivotal phase III trial showed that gemcitabine offers a survival advantage over a weekly bolus infusion of 5-FU, accompanied by an improved clinical benefit (Burris *et al*, 1997), the overall therapeutic results are still disappointing; the response rate was 5.4% with a clinical benefit response rate of 23.8% and a 1-year survival rate of 18% in patients treated with gemcitabine.

Therefore, a number of clinical studies have been undertaken to enhance the effectiveness of front-line chemotherapy. Despite promising results in early-phase clinical studies, the majority of newer approaches have failed to show clinically meaningful therapeutic advantages over the standard infusion of gemcitabine alone. Although regimens consisting of gemcitabine in combination with erlotinib or capecitabine have shown statistically significant increases in survival duration, the small amount of survival benefit and accompanying toxicities result in difficulties related to their translation into clinically meaningful improvements (Cunningham *et al*, 2005; Moore *et al*, 2007).

Considering the poor response rate (20% or less) of gemcitabine-based doublet treatment in the first-line setting, the short progression-free survival (PFS) (<4 months), and the increased use of gemcitabine as adjuvant treatment (Oettle *et al*, 2007), an additional problem in the therapeutic management of this common malignant disease, is the need for effective treatment alternatives in patients failing to respond to gemcitabine-based chemotherapy. To date, few studies have assessed second-line chemotherapy, primarily because of poor prognosis (Nakachi *et al*, 2007) and because of the limited life expectancy of those with advanced pancreatic cancer after failure of first-line chemotherapy (Kozuch *et al*, 2001; Tsavaris *et al*, 2005; Kulke *et al*, 2007; Xiong *et al*, 2008; Novarino *et al*, 2009). There is, therefore, a growing unmet need for a second-line chemotherapy regimen to treat patients with gemcitabine-refractory pancreatic cancer (Boeck and Heinemann, 2008; Kang and Saif, 2008).

The clinical benefit and safety of the FOLFIRI and FOLFOX regimens have been well established in a study of gastrointestinal cancer patients (Tournigand *et al*, 2004). In several phase II trials, irinotecan-based and oxaliplatin-based regimens have shown modest activity against advanced pancreatic cancer. A French group has reported that the FOLFIRI.3 regimen, composed of a split irinotecan infusion on days 1 and 3, with 5-FU for 2 days, showed promising activity in chemotherapy-naive and pre-treated patients with advanced pancreatic cancer. The confirmed response rate was 37.5%, with a median PFS of 5.6 months (Taieb *et al*, 2007). The study also suggested that there was no cross-resistance between gemcitabine and FOLFIRI.3 regimen. Furthermore, an oxaliplatin and 5-FU combination, at various doses and schedules, has been evaluated as second-line chemotherapy in pancreatic cancer patients after gemcitabine failure (Tsavaris *et al*, 2005; Gebbia *et al*, 2007; Novarino *et al*, 2009). Recently, a German group has reported that the 5FU/folinic acid (FA) plus oxaliplatin (OFF) regimen could prolong survival and improve the quality of life of advanced pancreatic cancer patients after gemcitabine failure compared with best supportive care alone with or without 5FU/FA (FF) (Oettle *et al*, 2005; Pelzer *et al*, 2008).

On the basis of these results, we conducted a randomised phase II study of the modified FOLFIRI.3 (mFOLFIRI.3) and modified FOLFOX (mFOLFOX) regimens as second-line treatments in patients with gemcitabine-refractory pancreatic cancer. The aim of this study was to select a better regimen, which should be investigated in future studies.

## Materials and methods

### Patients

Patients at least 18 years of age with histologically confirmed, locally advanced, or metastatic pancreatic adenocarcinoma, who were previously treated with gemcitabine-based first-line chemotherapy were eligible for this study if they met the following inclusion criteria: Eastern Cooperative Oncology Group (ECOG) performance status (PS) 0–2; measurable disease based on Response Evaluation Criteria in Solid Tumors (RECIST) criteria; no previous second-line chemotherapy; adequate bone marrow function, defined as a condition with leukocyte count >4000 per *μ*l, absolute neutrophil count >1500 per μl, haemoglobin >9.0 g per 100 ml, platelets >100 000 per μl; adequate renal and hepatic function, defined as a condition with serum creatinine <1.5 mg per 100 ml, bilirubin <1.5 mg per 100 ml (<2.5 mg per 100 ml in patients with obstructive jaundice and adequately decompressed bile duct obstruction), and serum transaminase <three-fold the upper normal limit (<five-fold the upper normal limit for patients with liver metastasis); adequate nutritional status, defined as a condition with albumin >3.0 g per 100 ml; and the giving of written informed consent. Patients were excluded if they had histology indicating a condition other than adenocarcinoma, brain metastasis, significant gastrointestinal bleeding or obstruction, any serious co-morbidity, axial skeletal radiotherapy within 6 months before study commencement, or peripheral neuropathy of grade 2 or worse. This study was initially approved by the Institutional Review Board of the Asan Medical Center. The study was conducted according to the tenets of the Declaration of Helsinki and guidelines on good clinical practice. The clinical trial registration number was NCT00786006.

### Study design and randomisation

This was an open-label, single-centre, randomised phase II trial using the two treatment arms of mFOLFIRI.3 and mFOLFOX. Random assignment was performed at a 1 : 1 ratio and patients were stratified by age (⩽65 years *vs* >65 years), ECOG PS (0–1 *vs* 2), and an earlier best overall response to gemcitabine (non-disease progression *vs* disease progression).

### Treatment dose and schedule

The mFOlFIRI.3 regimen consisted of irinotecan 70 mg m^−2^ (over 1 h) on day 1, leucovorin 400 mg m^−2^ (over 2 h) on day 1, 5-FU 2000 mg m^−2^ (over 46 h) from day 1, and irinotecan 70 mg m^−2^ (over 1 h) at the end of the 5-FU infusion every 2 weeks. The mFOLFOX regimen was composed of oxaliplatin 85 mg m^−2^ (over 2 h) on day 1, leucovorin 400 mg m^−2^ (over 2 h) on day 1, and 5-FU 2,000 mg m^−2^ (over 46 h) every 2 weeks. When haematologic or non-haematologic toxicities of grade ⩾2 occurred, chemotherapy was delayed until recovery to grade ⩽1. The doses of subsequent schedules were reduced by 25% in patients with grade ⩾3 haematologic and non-haematologic toxicities, and if toxicity was considered to be attributable, by the attending physician, to only one drug; the doses of other drugs were not modified. Treatment was continued until the occurrence of disease progression, unacceptable toxicity, or patient's refusal to continue. If disease progression was observed and patient performance was good, crossover to the alternate treatment arm was permitted.

### Pre- and on-treatment evaluation

Within 2 weeks before study enrolment, patients gave a complete medical history; underwent a full physical examination including ECOG PS; were sampled for a complete blood count, serum chemistry with electrolyte levels, a coagulation battery, and carbohydrate antigen 19–9 (CA 19–9) level; underwent urinalysis; underwent a chest X-ray; were assessed by electrocardiography; and were evaluated by computed tomography of the abdomen and pelvis (chest or any other region, if metastasis was suspected or previously detected). Before the administration of each cycle of chemotherapy, each patient was examined and reviewed for complete and differential blood counts and serum chemistry. More frequent review and monitoring were performed if clinically indicated. Tumour response was assessed every three cycles according to the RECIST criteria (Therasse *et al*, 2000). For each of these assessments, similar imaging techniques as used at baseline were used. The National Cancer Institute Common Terminology Criteria for Adverse Events, version 3.0, was used to assess toxicity.

### Statistical analysis

The primary end point was the 6-month survival rate. The randomised two-arm phase II design was used to select the more promising regimen of the two in terms of this criterion (Simon *et al*, 1985). Using this design, the regimen with the better survival rate is selected, irrespective of the difference between protocols. To permit at least a 90% probability of selecting a truly better regimen when the absolute difference in the 6-month survival rate was 15% or greater, 29 evaluable patients were needed in each arm. Survival time was calculated from the date of randomisation to the date of death from any cause. The secondary end points were overall response rate, PFS, overall survival (OS), and toxicity. Overall response rate was analysed on an intention-to-treat basis. PFS was defined as the time from randomisation to disease progression or death from any cause. PFS was censored at the date of the last visit for those patients who were alive without documented disease progression. OS and PFS were estimated by the Kaplan–Meier method. Patients were considered assessable if they had received at least two cycles of chemotherapy (over 4 weeks) and had at least one follow-up imaging study. However, patients were also considered assessable if they received less than two cycles because of rapid tumour progression. Survival curves were compared by the log-rank test. In multivariate analysis, Cox's proportional hazards model was used to identify independent prognostic factors for PFS and OS. All tests were two-sided and a *P*-value <0.05 was considered to be statistically significant. SPSS version 14.0 (SPSS, Chicago, IL, USA) was used for statistical analysis.

## Results

### Patient characteristics

From January 2007 to December 2008, 61 pancreatic cancer patients were enrolled at the Asan Medical Center, Seoul, Korea; 31 were randomly assigned to the mFOLFIRI.3 arm and 30 to the mFOLFOX arm. One patient in the mFOLFIRI.3 arm withdrew consent after the first cycle of chemotherapy and was lost to follow-up. Baseline characteristics were well balanced between the two treatment arms (Table 1). The median patient age was 55 years (range 35–73 years) and all but one patient was of ECOG PS 0 or 1. Twenty-one patients (34%) had undergone previous surgery and two (3%) had received palliative radiotherapy. Of the 16 patients who were prescribed adjuvant chemotherapy, gemcitabine was administered to three patients. Gemcitabine plus capecitabine was given to most patients (75%). After disease progression to a stage at which a salvage regimen was required, a crossover to the alternate protocol was undertaken by 12 patients (39%) in the mFOLFIRI.3 arm and by 7 (23%) in the mFOLFOX arm. The median time to crossover to the alternate treatment was 8.3 weeks (range 3.3–18.1 weeks) in the mFOLFIRI.3 arm, and 15 weeks (range 7.0–32.6 weeks) in the mFOLFOX arm.

### Primary end points

A total of 98 cycles of the mFOLFIRI.3 and 93 cycles of the mFOLFOX regimens were delivered with a median of 3 cycles (range 1–12 and 1–10 cycles, respectively) in both arms. With a median follow-up period of 24.4 weeks (range 0.8–40.8 weeks), 50 of 61 patients (82%) died. The 6-month survival rate was 27% in the mFOLFIRI.3 arm (95% confidence interval (CI)=13–46%) patients and 30% for those in the mFOLFOX arm (95% CI=15–49%). Except for two patients who died because of treatment-related complications, all deaths were attributable to disease progression *per se*.

### Secondary end points

The overall response rate values are listed in Table 2. Response evaluation was possible in 28 patients in the mFOLFIRI.3 arm and in 26 patients in the mFOLFOX arm. In the mFOLFIRI.3 arm, two patients could not be evaluated because of early death, and were lost to follow-up before the first response evaluation. In the mFOLFOX arm, response evaluation could not be achieved in four patients because of early death (two patients), loss to follow-up (one patient), and patient's refusal to continue with the trial (one patient). The overall response rate in the intention-to-treat population was 7% in the mFOLFOX arm (95% CI=1–22%). Overall response could not be ascertained in the mFOLFIRI.3 arm. The disease control rate (PR and stable disease) was 23% in the mFOLFIRI.3 arm (95% CI=11–40%) and 17% in the mFOLFOX arm (95% CI=7–34%).

**Figure 1:**
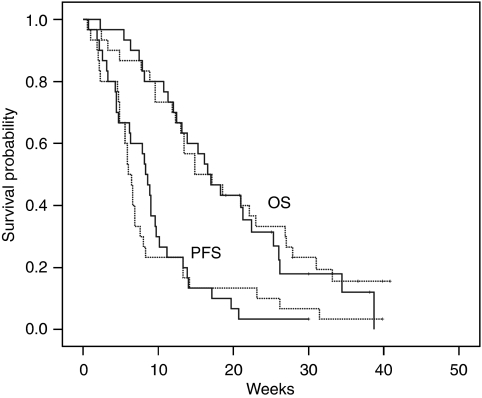
Survival curves for progression-free survival (PFS) and overall survival (OS). Modified FOLFIRI.3 (a regimen combining 5-fluorouracil, folinic acid, and irinotecan) is depicted as solid lines and modified FOLFOX (a regimen combining folinic acid, 5-FU, and oxaliplatin) as dotted lines.

The median PFS was 8.3 weeks for patients treated with mFOLFIRI.3 (95% CI=6.9–9.6 weeks) and 6.0 weeks for those given mFOLFOX (95% CI=5.1–6.9 weeks) (Figure 1). The median OS was 16.6 weeks for patients treated with mFOLFIRI.3 (95% CI=12.5–20.6 weeks) and 14.9 weeks for those given mFOLFOX (95% CI=8.0–21.8 weeks) (Figure 1). Turning to survival outcomes from the commencement of first-line chemotherapy, the median PFS was 34.9 weeks (95% CI=30.8–38.9 weeks) and 37.0 weeks (95% CI=32.0–42.0 weeks) for mFOLFIRI.3 and mFOLFOX, respectively. The median OS was identical at 47.1 weeks (95% CI=39.0–55.2 weeks and 36.0–58.3 weeks, respectively).

### Toxicity

The numbers of patients experiencing adverse events are presented in Table 3. In each treatment arm, 29 patients were available for toxicity assessment, and only two patients in the mFOLFOX arm were free from adverse events. The prevalence of severe toxicities was the same between the two regimens (38%); however, grade 3/4 asthaenia (3% *vs* 14%) developed more frequently in patients receiving mFOLFOX, whereas grade 3/4 diarrhoea (7% *vs* 0%) was more common in patients prescribed mFOLFIRI.3. Treatment-related mortality occurred in one patient in each group. One patient in the mFOLFIRI.3 arm died of septic shock complicated by febrile neutropaenia after 2 weeks of the first cycle. In one patient in the mFOLFOX arm, early death after the first cycle of chemotherapy was caused by severe pneumonia.

### Prognostic factors

In a univariate analysis of survival outcomes according to the clinical variables of all 60 patients (gender, age, ECOG PS, hypoalbuminaemia, anaemia, resectability at initial diagnosis, liver metastasis, and PFS under gemcitabine), hypoalbuminaemia (⩽3.5 mg 100 ml^−1^) and ECOG PS ⩾1 were significant prognostic factors for poor PFS and OS. In multivariate analysis, however, only hypoalbuminaemia predicted poor PFS (*P*=0.02, hazard ratio=1.97, 95% CI=1.14–3.39), but not OS.

## Discussion

Pancreatic cancer is well known to be refractive to chemotherapy and to show rapid progression. Until recently, patients with pancreatic cancer after gemcitabine-based chemotherapy failure have had little opportunity to receive second-line chemotherapy because of rapid performance deterioration (Nakachi *et al*, 2007; Kang and Saif, 2008). Therefore, few studies have focused on patients with advanced pancreatic cancer in a second-line setting. Moreover, as gemcitabine is known to be effective when used as adjuvant therapy, many patients who underwent curative resection received gemcitabine in this setting. This means that oncologists urgently require data on other chemotherapeutic options for gemcitabine-pretreated patients.

Gemcitabine plus oxaliplatin (GEMOX), oxaliplatin plus capecitabine (XELOX), capecitabine plus erlotinib, docetaxel plus gefitinib, and FOLFOX have been tested in gemcitabine-refractory pancreatic cancer patients and showed disease control rates of 19–53% and a median OS range of 2.9–6.7 months (Tsavaris *et al*, 2005; Demols *et al*, 2006; Kulke *et al*, 2007; Xiong *et al*, 2008; Brell *et al*, 2009; Novarino *et al*, 2009). Recently, another oxaliplatin-based regimen, 5-FU/FA plus oxaliplatin (OFF), was shown to offer significantly improved survival compared with 5-FU/FA (FF) in a phase III trial (CONKO 003) (Pelzer *et al*, 2008). In this randomised trial, including 160 gemcitabine-pretreated patients with advanced pancreatic cancer, patients receiving OFF achieved a median PFS of 13 weeks (*P*=0.012) and a median OS of 26 weeks (*P*=0.014), compared with 9 and 13 weeks, respectively, for FF-treated patients. However, there is no current consensus on optimal second-line therapy for gemcitabine-refractory advanced pancreatic cancer (Boeck and Heinemann, 2008; Kang and Saif, 2008). Both FOLFIRI.3 and FOLFOX have shown modest activity as first-line and second-line chemotherapy regimens (Tsavaris *et al*, 2005; Gebbia *et al*, 2007; Taieb *et al*, 2007; Novarino *et al*, 2009). We were also of the view that neither regimen showed significant cross-resistance to gemcitabine-based protocols (Gebbia *et al*, 2007; Taieb *et al*, 2007).

The results of this trial show that both combination regimens showed favourable efficacy and toxicity profiles in gemcitabine-pretreated patients with advanced pancreatic cancer. The 6-month survival rates were 27 and 30% and disease control rates were 23% and 17%, in patients treated with mFOLFIRI.3 and mFOLFOX, respectively. Of the 12 patients whose disease was controlled by these regimens, disease stabilisation was previously achieved in nine patients in gemcitabine-based regimens. The median PFS and median OS were 8.3 weeks and 16.6 weeks in the mFOLFIRI.3 arm, and 6.0 weeks and 14.9 weeks in the mFOLFOX arm, respectively. These were in line with the survival data of several previous studies (Tsavaris *et al*, 2005; Gebbia *et al*, 2007; Novarino *et al*, 2009).

Toxicities related to both regimens were quite expectable and generally manageable. Patients with toxicities of grade 3 or worse constituted 38% of each treatment arm. Common toxicities of both regimens included anaemia, neutropenia, asthaenia, nausea, vomiting, and mucositis. In accordance with the known toxicities of both regimens, diarrhoea developed more frequently in mFOLFIRI.3 arm patients and neuropathy was more common in those in the mFOLFOX arm. Although half the patients treated with mFOLFOX experienced peripheral neuropathy, this was mostly of grade 1. This may be related to a lower cumulative dose of oxaliplatin because of the early dropout caused by rapid disease progression. However, treatment-related mortality occurred in patients prescribed either regimen, and hence physicians need to guard against infectious complications in patients treated with these protocols.

Turning to prognostic factors affecting PFS and OS, hypoalbuminaemia, implying poor nutritional status, was a poor prognostic factor for PFS in this study. In contrast to a previous study (Herrmann *et al*, 2008), we could not find an association between the time to progression under first-line chemotherapy (⩽6 months) and PFS under second-line therapy, or residual survival. However, it is hard to draw conclusions with regard to this, because this study had small sample sizes, which might result in insufficient statistical power detecting significant prognostic factors.

Although this trial used adequate primary and secondary outcomes to represent the characteristics of the two regimens, the lack of assessment of clinical benefit or quality of life is a limitation of our study.

In conclusion, our trial not only showed that both mFOLFIRI.3 and mFOLFOX regimens could be safely used but also showed modest anti-cancer activities in gemcitabine-pretreated patients. Although further clinical trials are necessary for comparison with other regimens, these protocols may be reasonable therapeutic options in a second-line setting for patients with advanced pancreatic cancer, who were previously treated with gemcitabine-based chemotherapy.

## Figures and Tables

**Table 1:** Patient characteristics

**Characteristic**	**mFOLFIRI.3 (*n*=31) No. of patients (%)**	**mFOLFOX (*n*=30) No. of patients (%)**
*Age, median (range)*	55 (37–73)	55 (35–69)
<60 years	19 (61)	18 (60)
⩾60 years	12 (39)	12 (40)
		
*Gender*		
Male	24 (77)	20 (67)
Female	7 (23)	10 (33)
		
*ECOG PS*		
0	5 (16)	5 (17)
1	26 (84)	24 (80)
2	0 (0)	1 (3)
		
*Metastatic site*		
Liver	19 (61)	21 (70)
Peritoneum	19 (61)	11 (37)
Lung	6 (19)	5 (17)
Lymph nodes	15 (48)	14 (47)
Others	9 (29)	5 (17)
		
*Prior treatment*		
Surgery	10 (32)	11 (37)
Palliative radiotherapy	1 (3)	1 (3)
Adjuvant chemotherapy	7 (23)	9 (30)
Neoadjuvant chemoradiotherapy	0 (0)	1 (3)
		
*Prior gemcitabine-based regimen*		
Gemcitabine	4 (13)	2 (7)
Gemcitabine/capecitabine	20 (64)	26 (86)
Gemcitabine/erlotinib	4 (13)	2 (7)
Gemcitabine/cisplatin	3 (10)	0 (0)
		
*Previous response to gemcitabine-based regimen*		
CR	0 (0)	1 (3)
PR	10 (32)	9 (30)
SD	11 (35)	13 (43)
PD	10 (32)	7 (23)
		
*Survival at analysis*		
Alive	6 (20)	5 (17)
Dead	25 (81)	25 (83)
Crossover to alternative regimen	12 (39)	7 (23)

Abbreviations: ECOG PS=Eastern Cooperative Oncology Group performance status; CR=complete response; PR=partial response; SD=stable disease; PD=progressive disease.

**Table 2:** Overall response rate

**Overall Response**	**mFOLFIRI.3 No. of patients (%, 95% CI)**	**mFOLFOX No. of patients (%, 95% CI)**
PR	0 (0, 0–10)	2 (7, 1–22)
SD	7 (23, 11–40)	3 (10, 3–26)
PD	21 (68, 49–83)	21 (70, 52–84)
Not evaluable	3 (10, 3–26)	4 (13, 5–30)
Disease control	7 (23, 11–40)	5 (17, 7–34)

Abbreviations: PR=partial response; SD=stable disease; PD=progressive disease.

**Table 3:** Treatment-related toxicities

	**mFOLFIRI.3 no. of patients (%)**			**mFOLFOX no. of patients (%)**		
**Toxicity**	**G 1-2**	**G 3-4**	**All G**	**G 1-2**	**G 3-4**	**All G**
Anaemia	14 (48)	1 (3)	15 (52)	15 (50)	1 (3)	16 (55)
Neutropenia	6 (20)	7 (24)	13 (45)	8 (27)	6 (20)	14 (48)
Thrombocytopenia	3 (10)	1 (3)	4 (14)	9 (31)	1 (3)	10 (34)
Febrile neutropenia		1 (3)	1 (3)		0 (0)	0 (0)
Alopecia	3 (10)	0 (0)	3 (10)	0 (0)	0 (0)	0 (0)
Asthaenia	17 (58)	1 (3)	18 (62)	22 (76)	4 (14)	26 (90)
Diarrhoea	10 (34)	2 (7)	12 (41)	5 (17)	0 (0)	5 (17)
Anorexia	5 (17)	1 (3)	6 (21)	6 (21)	2 (7)	8 (28)
Nausea	12 (41)	1 (3)	13 (45)	13 (45)	1 (3)	14 (48)
Vomiting	6 (20)	3 (10)	9 (31)	11 (38)	3 (10)	14 (48)
Mucositis	8 (27)	1 (3)	9 (31)	8 (28)	2 (7)	10 (34)
Neurotoxicity	1 (3)	0 (0)	1 (3)	13 (44)	0 (0)	13 (45)
Maximum/patients^*^	18 (62)	11 (38)		16 (57)	11 (38)	

Abbreviation: G=grade.

^*^Maximum/patients, maximal toxicity in an individual patient.

The numbers of patients experiencing adverse events are listed.
